# Do acupuncture trials have lower risk of bias over the last five decades? A methodological study of 4 715 randomized controlled trials

**DOI:** 10.1371/journal.pone.0234491

**Published:** 2020-06-10

**Authors:** Youlin Long, Rui Chen, Qiong Guo, Shanxia Luo, Jin Huang, Liang Du

**Affiliations:** 1 Medical Device Regulatory Research and Evaluation Center, West China Hospital, Sichuan University, Chengdu, China; 2 Chinese Cochrane Center, West China Hospital, Sichuan University, Chengdu, China; 3 School of Clinical Medicine, Chengdu University of Traditional Chinese Medicine, Chengdu, China; 4 Department of Mental Health Center, West China Hospital, Sichuan University, Chengdu, China; 5 West China Hospital, Sichuan University, Chengdu, China; UNITED STATES

## Abstract

**Objective:**

To evaluate the change of the risk of bias (RoB) of acupuncture randomized controlled trials (RCTs) in the past five decades.

**Methods:**

Multiple databases were searched. We included RCTs identified from systematic reviews (SRs) on acupuncture. General characteristics and RoB judgment for each domain were extracted. The proportions of RCTs at high and unclear RoB were calculated and the changes were examined by the Mann-Kendall test.

**Results:**

We included 368 SRs including 4 715 RCTs. The rates of RCTs at unclear RoB were the highest in allocation concealment (63%), and at the lowest in incomplete outcome data (35%); in the last five decades, statistically significant reductions were found for random sequence generation (*P* < 0.001) and selective reporting (*P* = 0.01), and increases for blinding of participants and personnel (*P* < 0.001), blinding of outcome assessment (*P* < 0.001) and incomplete outcome data (*P* = 0.04). For the proportions of RCTs at high RoB, blinding of participants and personnel (47%) and blinding of outcome assessment (35%) were the poorest domains; there were no significant differences in changes for all domains.

**Conclusions:**

Although improvements concerning unclear risk were observed for random sequence generation and selective reporting, major issues remain for allocation concealment and blinding. It is imperative to use valid randomization, specify how it is conducted, and try to test for selection bias and the success of masking by using the Berger Exner test.

## Introduction

The Grading of Recommendations Assessment, Development and Evaluation (GRADE) system was often used to evaluate the quality of evidence [1]. Due to the lack of high-quality evidence from systematic reviews (SRs) on acupuncture, the recommendations for acupuncture in the guidelines are not high in strength [2]. Several studies have shown that a high risk of bias (RoB) was the main reason for rating down the quality of evidence [3–5], and the conclusion cannot be trusted if randomized controlled trials (RCTs) were rated as high RoB [6]. Additionally, one methodological study has shown that supplementary information from trialists can significantly increase the rate of trials at low RoB rather than high RoB by reducing the rate of unclear RoB [7]. Therefore, it is vitally important to reduce the proportion of RCTs having unclear or high RoB in the acupuncture field.

RoB of RCTs included selective reporting, failure to perform randomization, allocation concealment, blinding, and failure to consider the intention-to-treat principle for loss to follow-up [8]. Such limitations may lead to an underestimation or overestimation of the true effects of interventions [9]. Another concern about the reporting should be noted. RoB should be rated as unclear if there is a lack of information to give a final judgment of low or high RoB [10]. The proportions of RCTs considered by the systematic reviewers to be at unclear and high RoB could be used as surrogates for poor reporting and inadequate methods, respectively [11].

Many studies have assessed the RoB or reporting quality in acupuncture RCTs, but they are restricted to the number of RCTs included, specific topics, journals, or countries [12–17]. As far as we know, there has not been an article that assesses the RoB of acupuncture RCTs from the inception of research. An assessment of this will provide valuable information about the strengths and limitations for RoB in acupuncture RCTs over time and enable targeted efforts in improving the conduct and reporting in the future.

Consequently, we aimed to explore the changes of RoB of acupuncture RCTs over time, and propose solutions with regards to what actions could be undertaken to reduce the high or unclear RoB of acupuncture RCTs.

## Methods

### Inclusion and exclusion criteria

Eligible SRs must determine the effect of acupuncture and include RCTs. Acupuncture is a treatment procedure requiring filiform needles penetrating the acupoint with adequate manipulation, such as body acupuncture or abdominal acupuncture [18]. We only considered SRs that included a final judgment of the RoB for at least one domain of the Cochrane RoB tool: random sequence generation, allocation concealment, blinding (blinding overall, blinding of participants and personnel, or blinding of outcome assessment), incomplete outcome data and selective reporting. Additionally, we only included SRs that had two or more independent judgments for RoB. We excluded SRs with acupuncture combined therapy and conference abstracts.

### Search strategy

PubMed, Embase, China National Knowledge Infrastructure (CNKI), Chinese WanFang Database, and China Science and Technology Journal Database (VIP) were searched from their inception to 24 November, 2017. We used a comprehensive search strategy, including a combination of text words and Medical Subject Headings (MeSH) terms and related to acupuncture and SR. Search strategies in PubMed are shown in S1 Table. The screening process was not limited by publication status.

### Selection of eligible SRs and RCTs

Two trained researchers independently screened the titles and abstracts of all the articles. Subsequently, full texts of potential articles were further examined to determine eligibility. Disagreements were resolved by discussion. For RCTs included in more than one SR, considering the learning curve for authors for using the RoB tool, we only considered the RoB judgment in the most recent SR [9, 11]. Consequently, we kept one of the duplicate RCTs. To identify the duplicate RCTs, the references of SRs were first identified. Then, citations of RCTs were managed in EndNote X8 and “Find duplicates” was performed. Finally, we checked whether the identified RCTs were the same ones, and manually identified duplicate RCTs in the remaining citations.

### Data extraction and standardization

Microsoft Access 2016 software was used to develop a standard data extraction table. Two reviewers independently exacted the data, and disagreements were resolved by consensus of them. The following data in SRs were extracted: first author, disease, primary intervention, primary outcomes, publication year (SR and RCT), RoB domains, and final RoB judgments. According to the Cochrane Handbook [10], systematic reviewers should evaluate the blinding of outcome assessment and incomplete outcome data for each outcome. Consequently, if the SRs reported more than one judgment for the same domain above, we extracted the RoB judgments corresponding to subjective outcomes, due to the potentially higher risk of subjective outcomes [9]. For domain blinding overall, we regarded the RoB as the same for both blinding of participants, and personnel and blinding of outcome assessors. For duplicate RCTs, we extracted the RoB judgment in the SR for the most recent.

Because the wording of RoB domains varied across SRs, two authors independently standardized all relevant terms according to the Cochrane Handbook [10]. Additionally, two researchers also independently standardized the classification of RoB assessment in terms of low, high or unclear RoB due to the inconsistent classification of judgments in SRs, such as yes, no, or unclear. These inconsistent classifications were only expressed in different ways but had the same meaning. All disagreements were resolved by discussion.

### Data analysis

Microsoft Excel 2019 was used to calculate the proportions of RCTs at high and unclear RoB in total. We conducted the non-parametric Mann-Kendall test to assess the monotonically increasing or decreasing trends across the study years using R software (version 3.6.3), including the changes of the annual number of RCTs, and the proportion of RCTs at high or unclear risk.

## Results

### Selection and general characteristics

**Fig 1:**
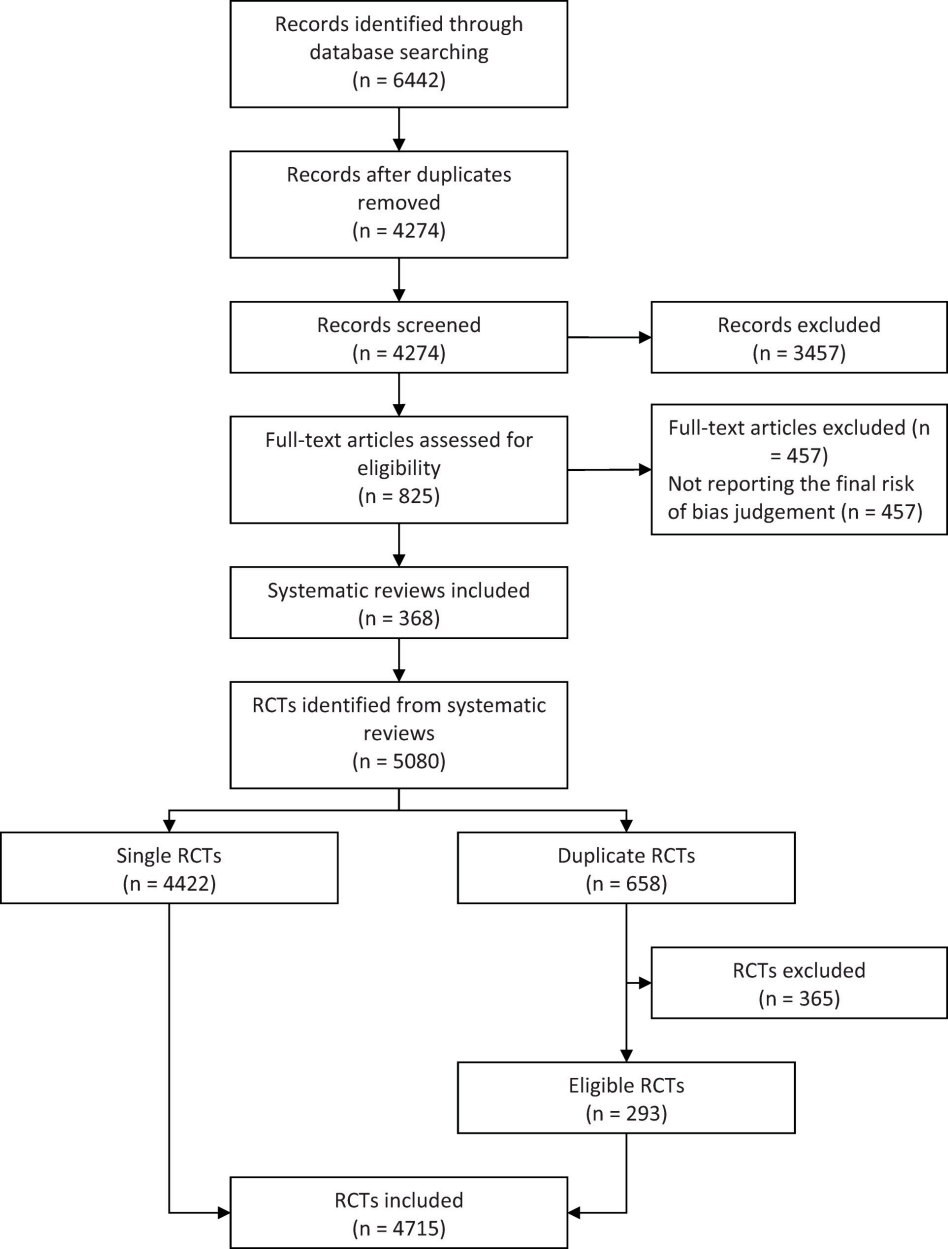
PRISMA flow diagram.

The selection process is presented in Fig 1. We retrieved 825 acupuncture SRs, with RoB assessment performed in 368 (45%) SRs. A total of 4 715 RCTs were included in these 368 SRs. Of these RCTs, 962 (20%) focused on pain, and 467 (10%) focused on sequelae of stroke (Table 1). The primary interventions were traditional acupuncture in 3899 (83%) RCTs and electroacupuncture in 816 (17%) RCTs. The top 3 outcomes were effective rate (45%), rate of adverse events (9%), and pain intensity improved (6%). The annual number of RCTs included in this study indicated an upward trend from 1974 to 2011 and has declined since 2011 (S1 Fig). Overall, the number of RCTs increased significantly in the last five decades (z = 6.56, *P* < 0.01).

**Table 1:** General characteristics of included RCTs of acupuncture (n = 4 715).

Characteristics	n (%)
10 highest represented disease	
Pain	962 (20%)
Sequelae of stroke	467 (10%)
Insomnia	213 (5%)
Cerebral infarction	169 (4%)
Stroke	137 (3%)
Obesity	122 (3%)
Parkinson	89 (2%)
Depression	83 (2%)
Irritable bowel syndrome	78 (2%)
Acne	75 (2%)
Primary intervention	
Traditional acupuncture	3899 (83%)
Electroacupuncture	816 (17%)
10 highest represented primary outcomes	
Effective rate	2111 (45%)
Rate of adverse events	439 (9%)
Pain intensity improved	264 (6%)
Visual analog pain scale	220 (5%)
Cure rate	219 (5%)
Recurrence rate	201 (4%)
Modified Neurological Severity Scores	163 (3%)
Activity daily living scores	159 (3%)
Disability	121 (3%)
Mortality	121 (3%)
Publication year, median (interquartile range)	2009 (2005–2012)

### Overall assessment of poor reporting and inadequate methods

**Fig 2:**
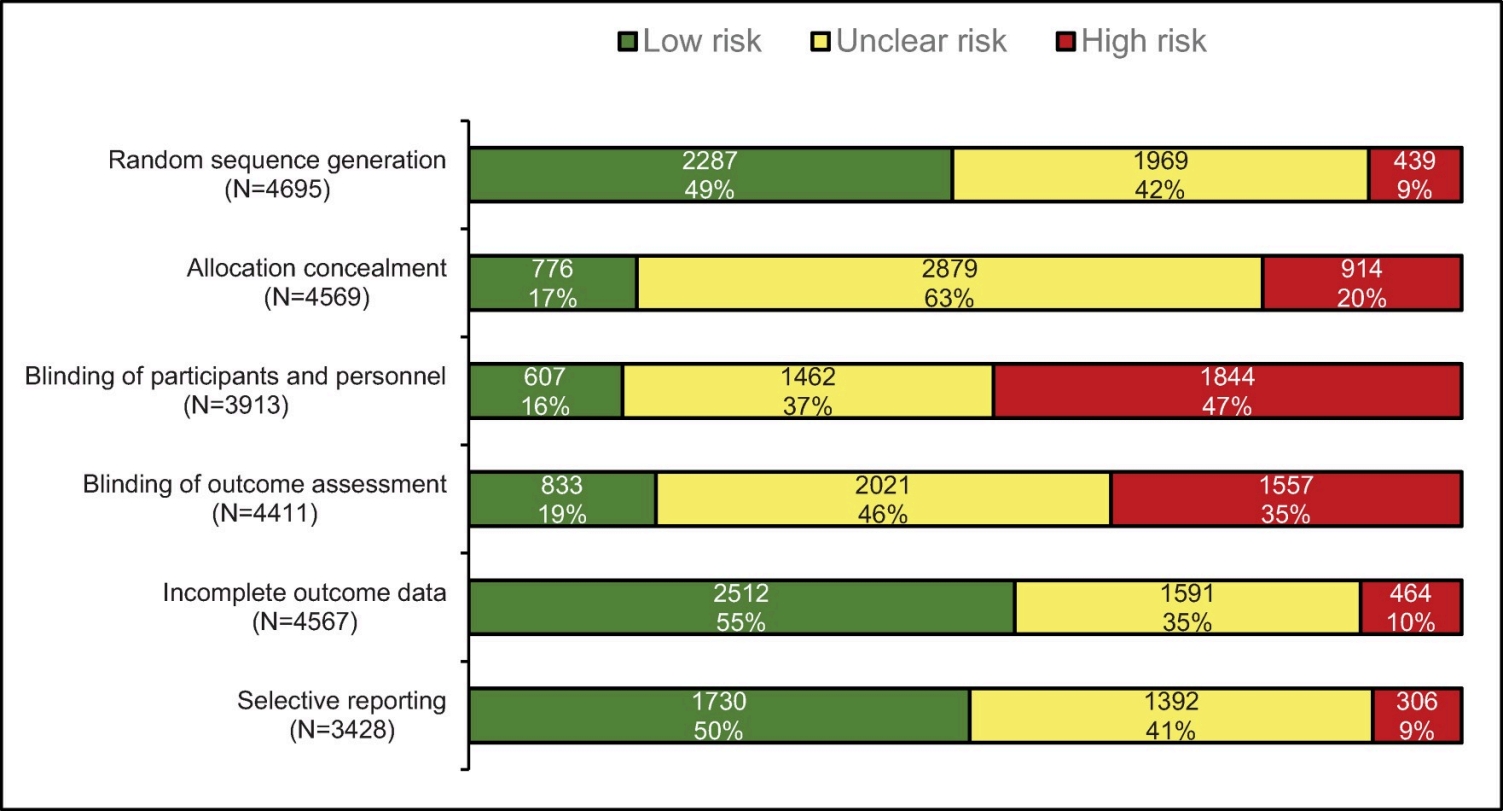
RoB for each domain in 4 715 RCTs on acupuncture.

The proportion of RCTs at unclear RoB was 35% and 37% of all RCTs for incomplete outcome data and blinding of participants and personnel, but was 63% for allocation concealment, and 46%, 42% and 41% for blinding of outcome assessment, random sequence generation, and selective reporting, respectively (Fig 2). The proportion of RCTs at high RoB was low for random sequence generation, selective reporting and incomplete outcome data (9%, 9%, and 10%, respectively), and higher for allocation concealment, blinding of outcome assessment and blinding of participants and personnel (20%, 35%, and 47%, respectively) (Fig 2).

### Temporal trends of risk of bias

#### Evolution of high risk

**Fig 3:**
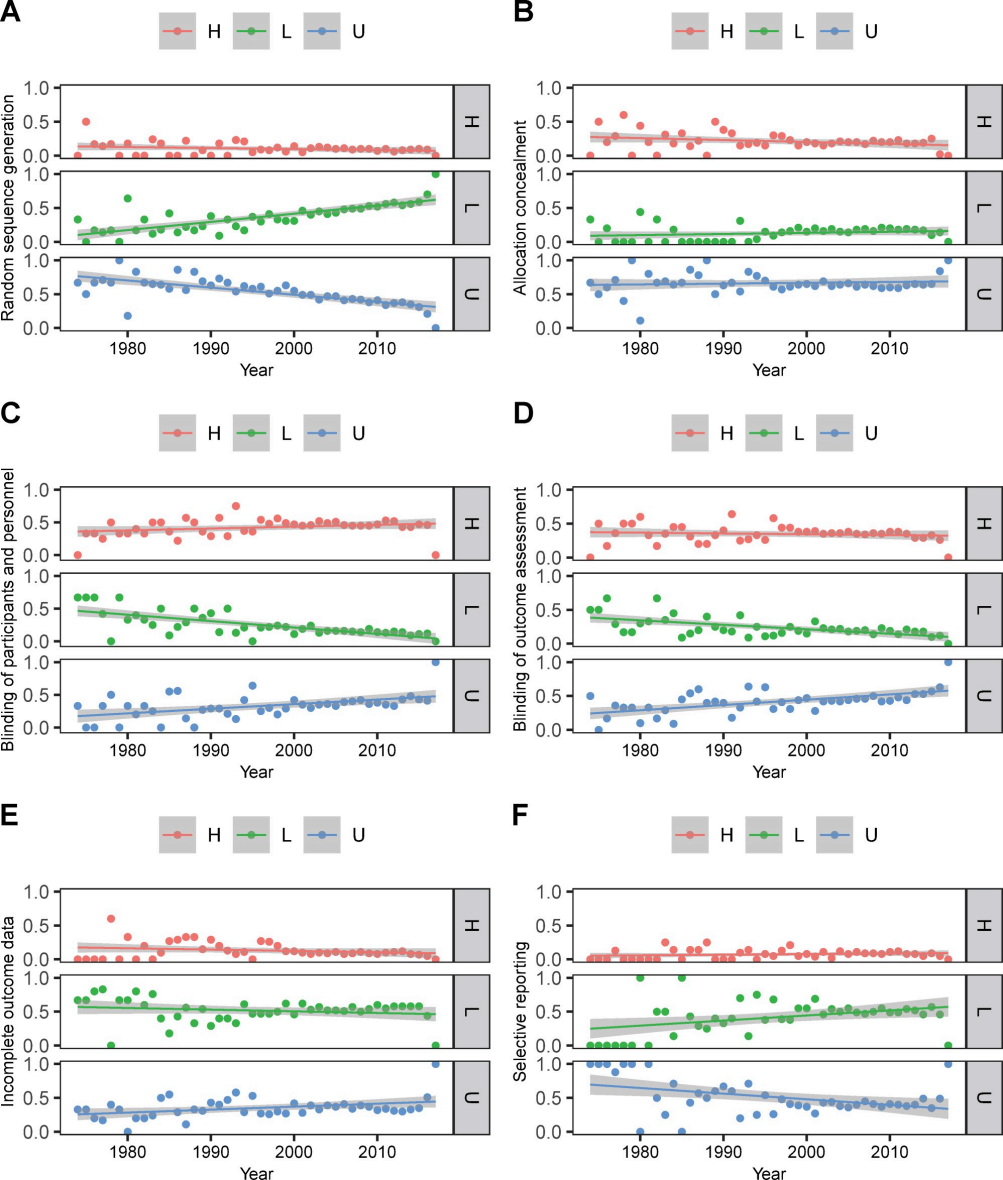
Evolution of poor reporting over time in 4715 RCTs on acupuncture.

Over the past five decades, there seems to be a slight decrease in the proportion of RCTs at high risk which included random sequence generation, allocation concealment, blinding of outcome assessment, and incomplete outcome data, but these differences were not statistically significant (z < 0, *P* > 0.05, Fig 3 and Table 2). Although a slight increase in the proportion of RCTs at high risk was observed for blinding of participants and personnel and selective reporting, there were no significant differences of changes (z > 0, *P* > 0.05).

**Table 2:** Results of the Mann-Kendall test for the temporal trends of risk of bias.

Domains	Judgment	z	*P*-value
Random sequence generation	High risk	-0.79	0.43
Allocation concealment	High risk	-1.20	0.23
Blinding of participants and personnel	High risk	1.78	0.08
Blinding of outcome assessment	High risk	-1.15	0.25
Incomplete outcome data	High risk	-1.41	0.16
Selective reporting	High risk	1.35	0.18
Random sequence generation	Unclear risk	-6.43	< 0.01
Allocation concealment	Unclear risk	-0.39	0.69
Blinding of participants and personnel	Unclear risk	3.85	< 0.01
Blinding of outcome assessment	Unclear risk	4.33	< 0.01
Incomplete outcome data	Unclear risk	2.08	0.04
Selective reporting	Unclear risk	-2.55	0.01

#### Evolution of unclear risk

A statistically significant reduction in the proportion of RCTs at unclear risk was observed for random sequence generation (z = -6.43, *P* < 0.01, Fig 3 and Table 2) and selective reporting (z = -2.55, *P* = 0.01). On the contrary, the proportion of RCTs at unclear risk increased with statistically significant trends for blinding of participants and personnel (z = 3.85, *P* < 0.01), blinding of outcome assessment (z = 4.33, *P* < 0.01) and incomplete outcome data (z = 2.08, *P* = 0.04). Additionally, for allocation concealment, the change was not statistically significant (z = -0.39, *P* = 0.69).

## Discussion

This methodological study included a great number of RCTs from 368 acupuncture SRs involved in a broad range of topics. We found great improvements in the proportions of RCTs at unclear risk for random sequence generation and selective reporting. However, our results raise concerns about allocation concealment and blinding. For allocation concealment, more than half (63%) of RCTs were rated as unclear RoB, and the rates of RCTs at unclear and high risk did not change over time. More importantly, the proportions of RCTs at unclear and high risk continue to increase for blinding of participants and personnel and blinding of outcome assessment.

Due to a lack of sufficient details, the unclear RoB of RCTs was serious for all the domains, especially allocation concealment. Most studies did not mention allocation concealment in the articles or not have detailed reporting, such as sealed opaque envelopes or central random methods [17]. A large proportion of RCTs was rated as high RoB for blinding, and one possible explanation for this was that blinding of acupuncturists is infeasible in RCTs due to the nature of acupuncture intervention [19]. Additionally, it is difficult for acupuncture-experienced participants to be blinded due to the general recognition of acupuncture. Although some studies performed the blinding of participants or outcome assessors, they failed to report the specific process or clarify who was blinded [17, 20]. For random sequence generation, this study may overestimate the number of RCTs at low RoB. According to the Cochrane Handbook version 5.1.0 [10], trials reporting “using a computer random number generator”, “envelopes” or “minimization” could be judged as low RoB. However, Berger and colleagues [21, 22] have argued that “using a computer random number generator” is not a specific randomization method (it should be judged as unclear RoB), and “envelopes” and “minimization” were flawed method (they should be judged as high RoB).

Our findings are similar to those previously reported, in which the authors found the reporting in acupuncture RCTs for a broad range of topics was generally poor when CONSORT and STRICTA were used to assess the reporting quality [12–14, 23–31]. Additionally, several studies have shown the overall high rates of RCTs at unclear and high RoB on acupuncture, which are consistent with our findings [16, 20, 32]. Kim assessed the methodological quality of acupuncture RCTs in the Korean literature, and they found that a low proportion of trials reported allocation concealment, participant blinding and outcome assessment blinding, which agrees with our results [15]. Previous studies have shown that reporting quality of acupuncture trials has improved since the introduction of the CONSORT and STRICTA in China, but the rates of some important items were still low [33, 34].

This study has strengths. To our knowledge, our study is the first that explores the evolution of RoB of RCTs in the whole of the acupuncture field. Additionally, we used a large sample of RCTs coving multiple topics (n = 4715) to support our conclusions. Finally, we used a comprehensive search strategy and two researchers independently screened citations and extracted data to follow the scientific process and minimize potential bias.

Several limitations were noted. First, our results relied on the RoB assessments from a cohort of acupuncture SRs rather than evaluating by ourselves. Consequently, we only included SRs that at least two independent assessors performed the RoB assessment to ensure the reliability of our results. Second, RCTs included in acupuncture SRs may fail to represent the real acupuncture RCTs, but we think it is unlikely to include such a significant number of RCTs if we did not take advantage of SRs. Third, we excluded SRs involved in combined therapy. It limited the applicability of these findings. Fourth, we focused on the internal validity/ risk of bias but did not focus on other reasons for the weak reliability in RCTs, such as the skill of doctors, doctor-patient relationship, or ridiculous assumptions in the statistical analyses. Finally, most SRs included the RCTs published one year before the publication date of SRs, and our study may miss the latest information of RCTs. Our results are from a large sample of acupuncture trials with a long span, so we believe there is no reason why this trend would have changed recently. This study is part of a larger project, the next step of which is to explore the impact of characteristics on RoB and the impact of RoB on treatment effect sizes of acupuncture meta-analyses according to the method of meta‐epidemiological study [35].

Our study shows acupuncture RCTs have room for improvement in reducing high RoB. Valid randomization procedures, such as the big stick, Chen, or maximum tolerated imbalance, should be used [22]. Although blinding is not always feasible, as far as possible trialists should blind the outcome assessors, and blinding of participants and personnel should be encouraged in the explanatory RCTs. When using blinding to control for the psychological effects, the key challenge is how to ensure that participants cannot distinguish the real acupuncture from a sham control [36]. Additionally, non-insertion and needle-insertion sham acupuncture can be combined as a control to improve the effect of blinding [37]. Chen recommended using a pilot study to validate the effect of blinding before the commencement of the formal RCTs [19]. Berger-Exner test was developed to directly detect selection bias and the success of masking [38, 39]. Including Berger–Exner test results in final trial reports should become standard practice [22].

It is also essential to improve the reporting quality in acupuncture RCTs. First, trialists should take on responsibility for following the CONSORT and SRTICTA statement [40, 41], and involve methodologists to improve the design and reporting. Second, journals must promote the implementation of reporting guidelines, such as submission of the checklists with the manuscript. Third, peer reviewers could identify the problems in conduct and reporting. Finally, guideline developers need to give more sensitive keywords related to allocation concealment and blinding.

## Conclusion

Although improvements concerning unclear risk were observed for random sequence generation and selective reporting over time, major issues remain in the unclear or high RoB for allocation concealment and blinding. It is imperative to use valid randomization, specify how it is conducted, and try to test for selection bias and the success of masking by using the Berger Exner test in RCTs on acupuncture.

## Supporting information

S1 Checklist PRISMA 2009 checklist. (DOC)

S1 Table Search strategy in PubMed. (DOCX)

S1 Fig The number of RCTs included in acupuncture SRs from 1974 to 2017. (EPS)
