# Supplementary material for: Do acupuncture trials have lower risk of bias over the last five decades? A methodological study of 4 715 randomized controlled trials
Source: PLoS One. 2020 Jun 10;15(6):e0234491. doi: 10.1371/journal.pone.0234491 (PMC7286517; doi:10.1371/journal.pone.0234491)
Supplement: S1 Table — (DOCX) [file pone.0234491.s002.docx]

**S1 Table. Search strategy in PubMed.**

| 1 | "systematic review"[Text Word] |
| --- | --- |
| 2 | meta-analysis[Publication Type] |
| 3 | 1 OR 2 |
| 4 | acupuncture[MeSH Terms] |
| 5 | acupuncture[Text Word] |
| 6 | electroacupuncture[MeSH Terms] |
| 7 | electroacupuncture[Text Word] |
| 8 | 4 OR 5 OR 6 OR 7 |
| 9 | 3 AND 8 |
